# Supplementary figures and images for: Immunoprofiling of fresh HAM/TSP blood samples shows altered innate cell responsiveness
Source: PLoS Negl Trop Dis. 2021 Nov 12;15(11):e0009940. doi: 10.1371/journal.pntd.0009940 (PMC8631667; doi:10.1371/journal.pntd.0009940)

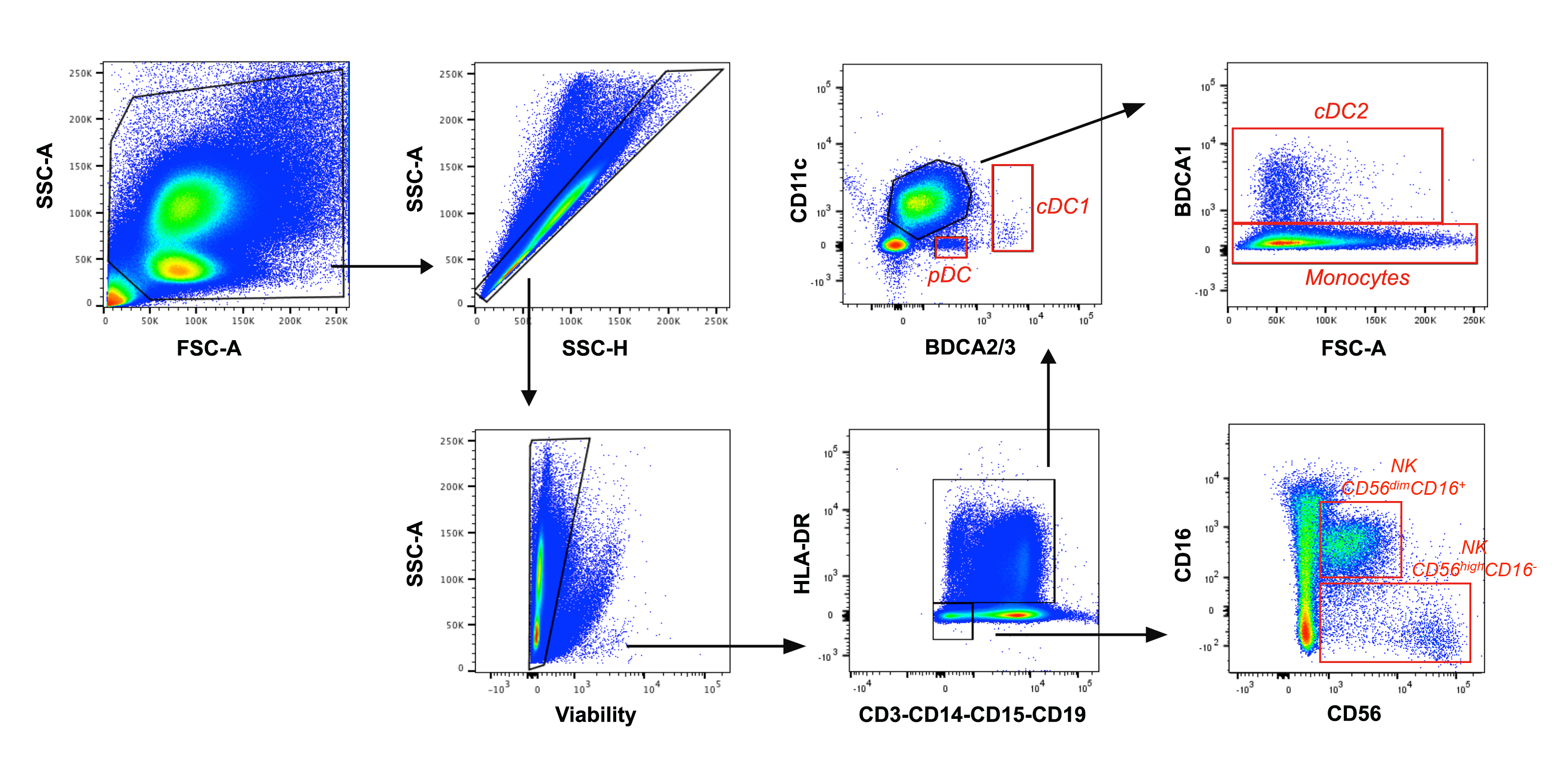

Supplement: S1 Fig — Flow cytometry collected datasets were analyzed with FlowJo software. A total of 2x10^6^ cells were registered and selected by cell size and granularity. After selection of single cells, viable cells were gated and innate immune cell populations were identified as indicated. (TIF) [file pntd.0009940.s001.tif]

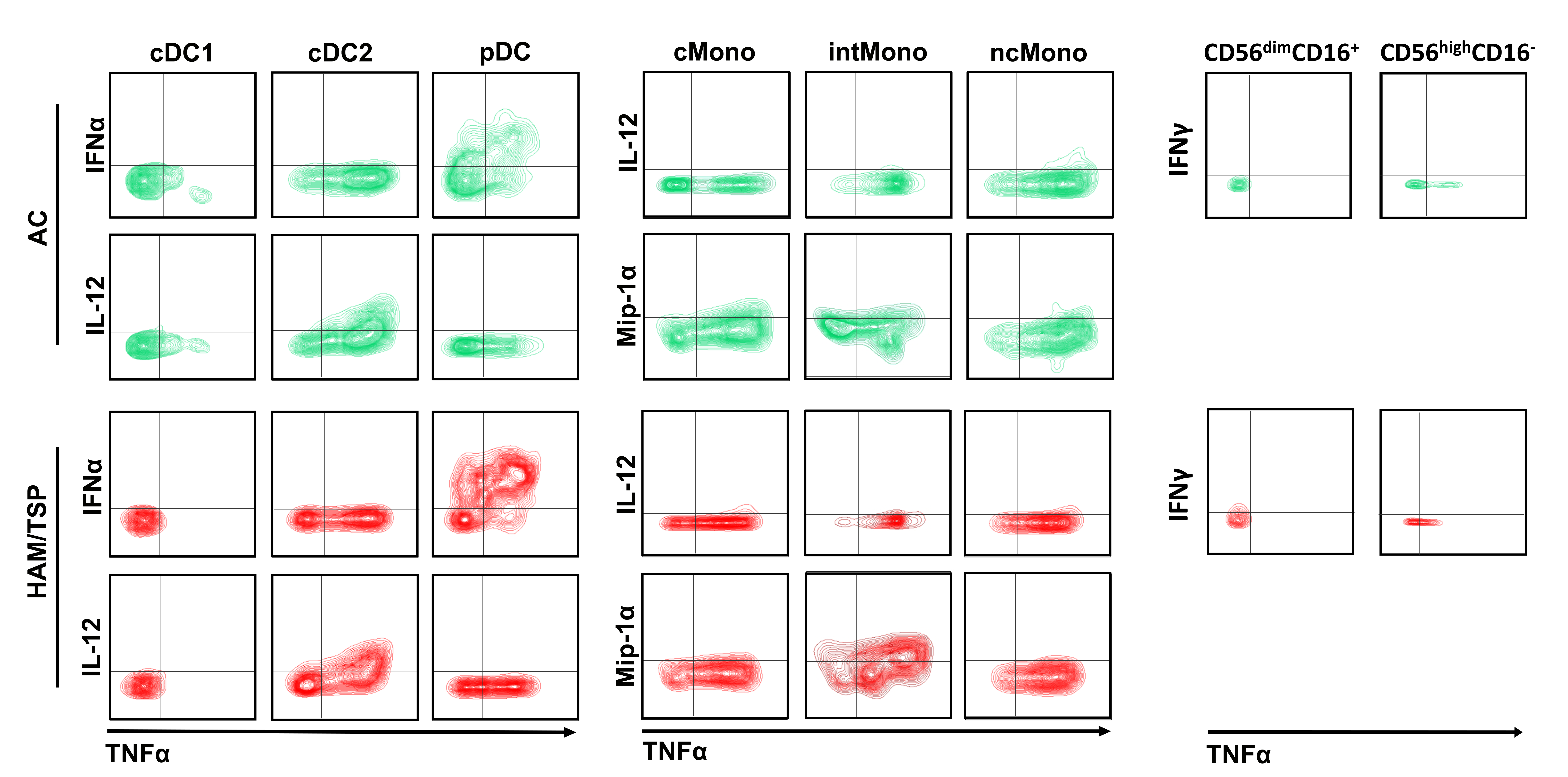

Supplement: S2 Fig — Example of the gating strategy for cytokine determination in AC and HAM/TSP group for IFNα, IL-12 MIP-1α and TNFα in the different cell subsets. (TIF) [file pntd.0009940.s002.tif]

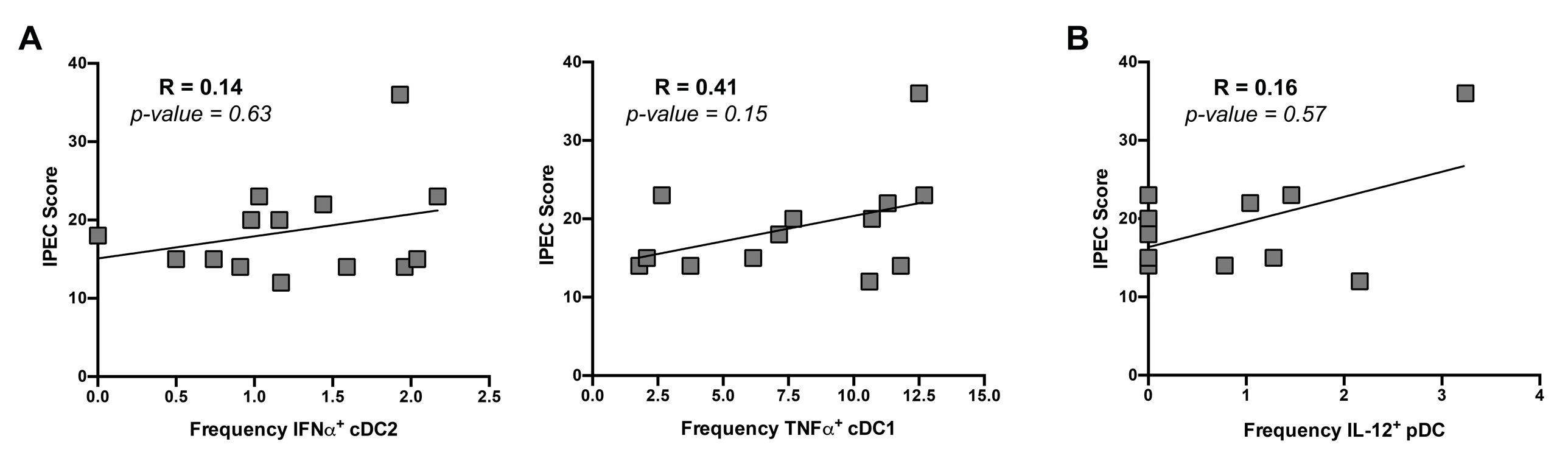

Supplement: S3 Fig — Correlation between IPEC Score of HAM/TSP patients and the frequency of (A) IFNα+ cDC2 and TNFα+ cDC1; and (B) IL-12+ pDC after R848 stimulation. Spearman test was applied to determine the correlation between the two factors. (TIF) [file pntd.0009940.s003.tif]

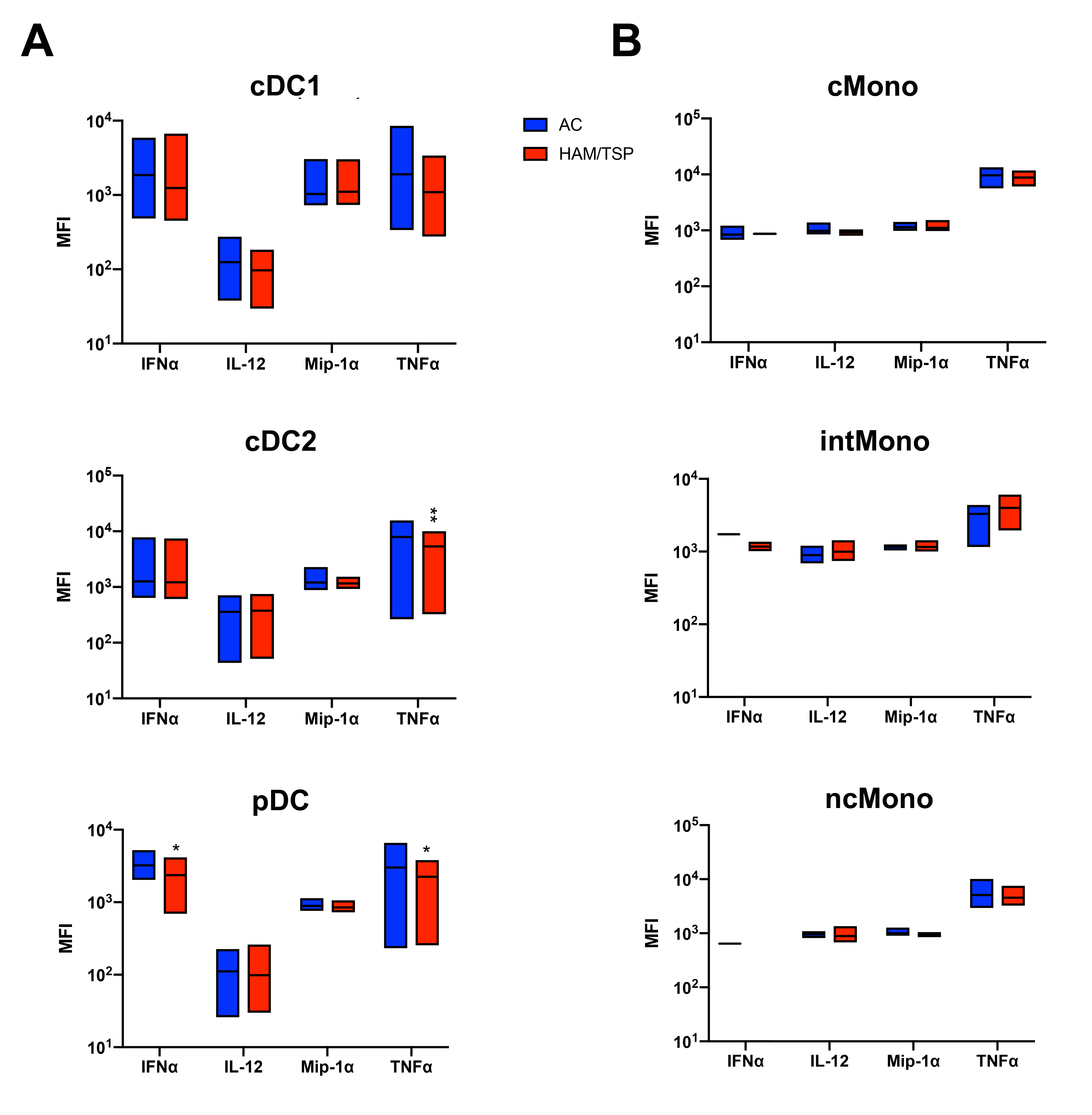

Supplement: S4 Fig — Median intensity fluorescence (MFI) of the cytokine expression for dendritic cell (A) and monocytes (B) subsets after TLR7 stimulation in AC and HAM/TSP patients. One-way ANOVA followed by Sidak’s correction for multiple comparisons was applied. (TIF) [file pntd.0009940.s004.tif]

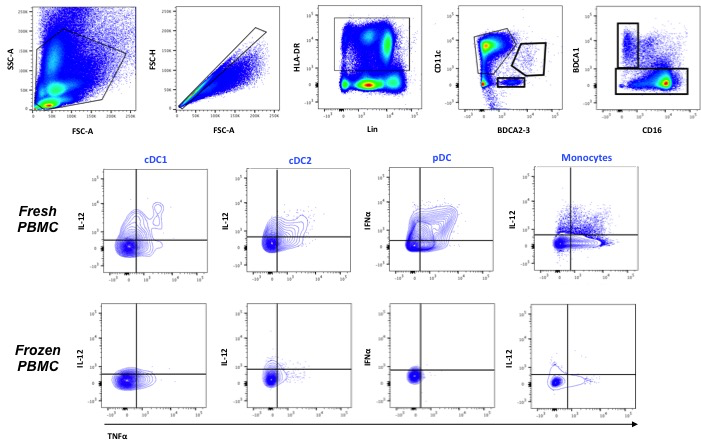

Supplement: S5 Fig — A. A total of 2x10^6^ cells from fresh or frozen PBMCs were registered and selected by cell size and granularity. After selection of single cells, viable cells were gated and innate immune cell populations were identified as indicated in S1 Fig. B. PBMCs from fresh or frozen PBMCs were stimulated with R848 and analyzed by flow cytometry for their intracellular production of IL-12 and TNFα (for BDCA3+ cDC1; BDCA1 cDC2 and monocytes) or IFNα and TNFα (for pDC). (TIF) [file pntd.0009940.s005.tif]

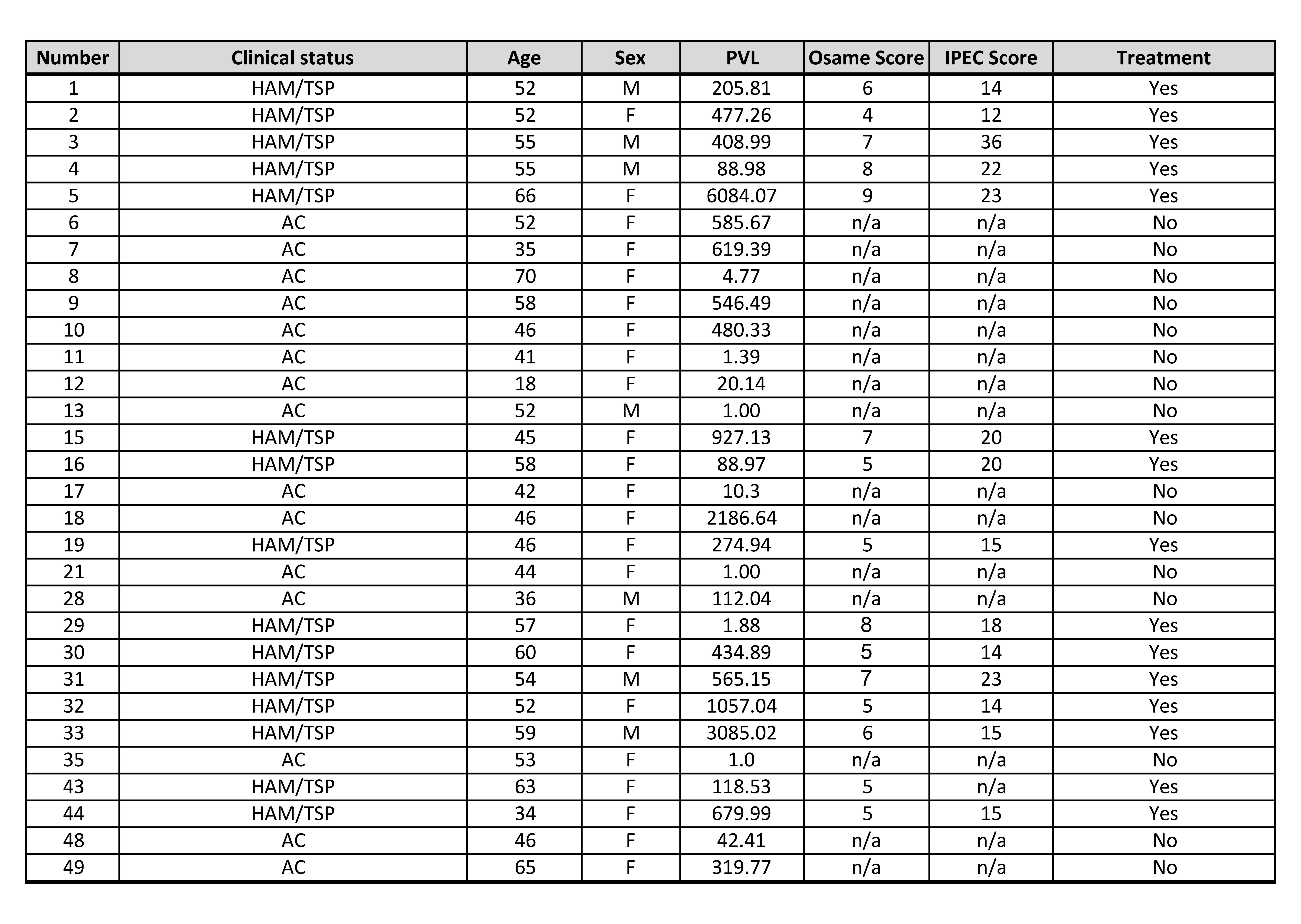

Supplement: S1 Table — Clinical status, sex, age, PVL, motors score and treatment information is detailed for each HTLV-1-infected subjects enroller in the study. (TIF) [file pntd.0009940.s006.tif]

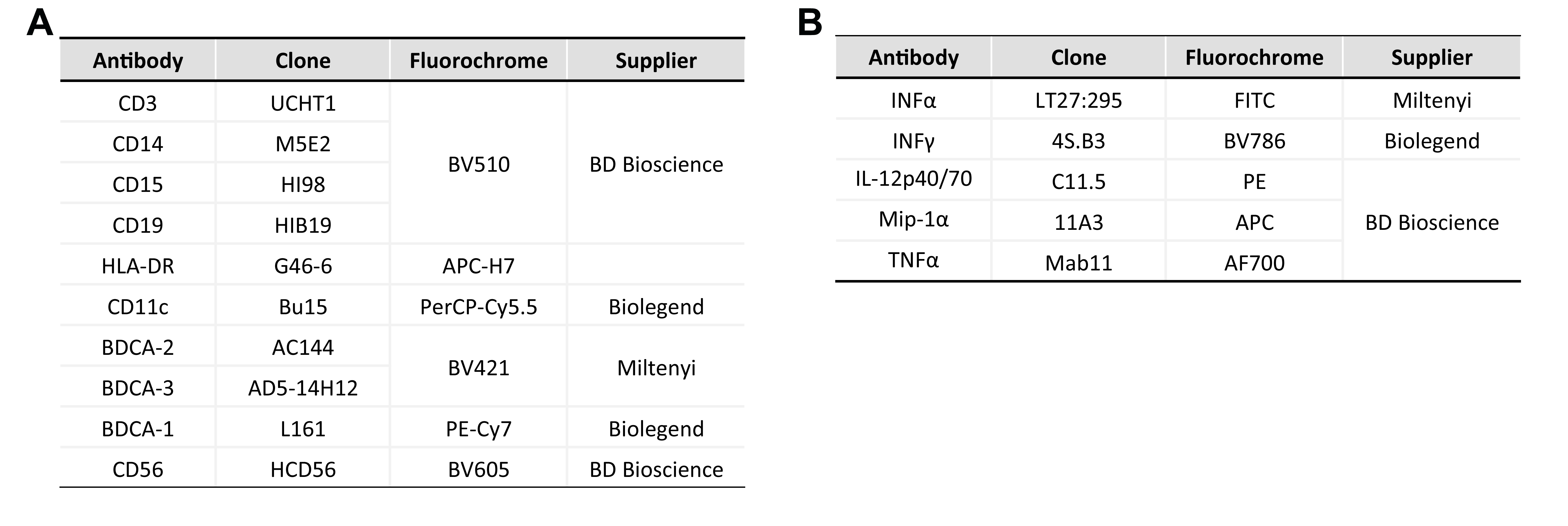

Supplement: S2 Table — Recapitulative list of the (A) membrane markers antibodies and (B) intracellular markers antibodies used for the analysis of the innate immune response by flow cytometry. (TIF) [file pntd.0009940.s007.tif]

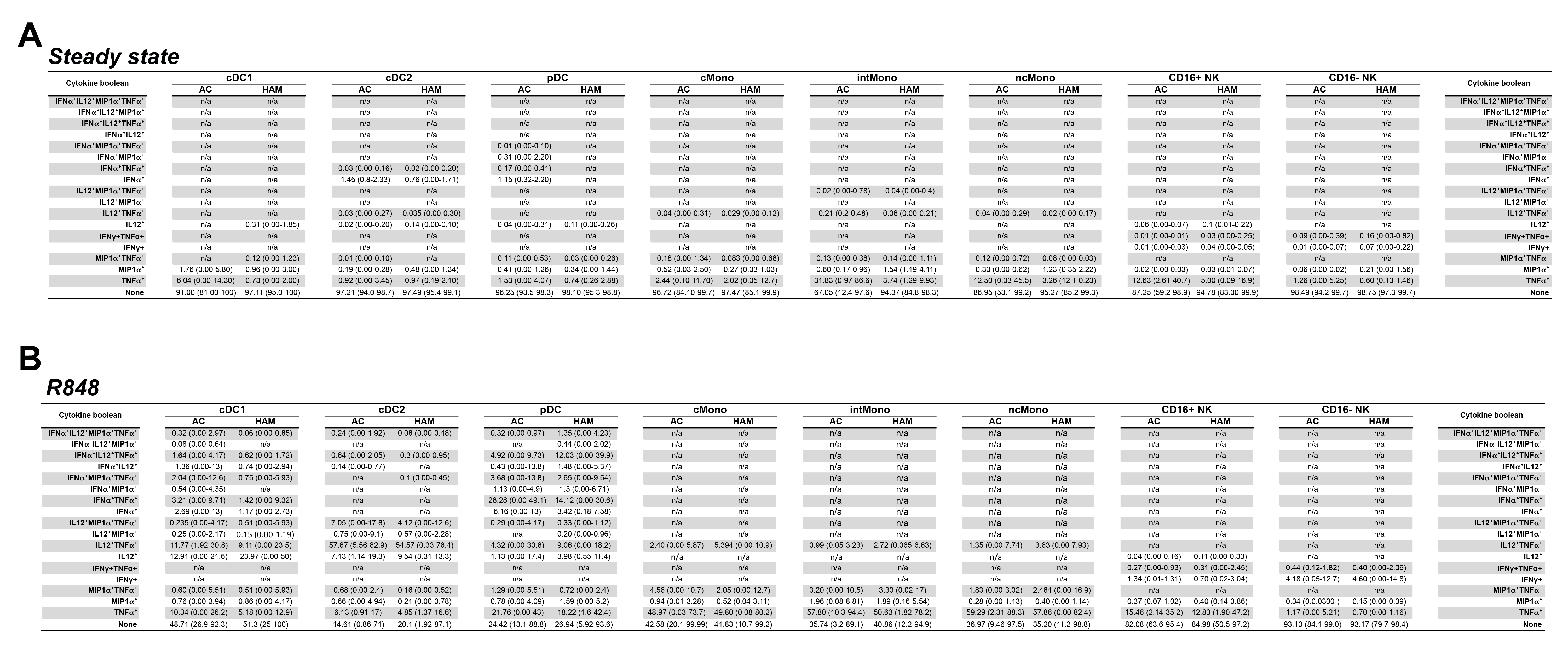

Supplement: S3 Table — Cell frequency of multi-cytokine production determined using boolean analysis at (A) steady state and (B) after TLR7/8 stimulation. (TIF) [file pntd.0009940.s008.tif]
