# Supplementary material for: Immunoprofiling of fresh HAM/TSP blood samples shows altered innate cell responsiveness
Source: PLoS Negl Trop Dis. 2021 Nov 12;15(11):e0009940. doi: 10.1371/journal.pntd.0009940 (PMC8631667; doi:10.1371/journal.pntd.0009940)
Supplement: Attachment — Submitted filename: rebutal letter.docx [file pntd.0009940.s009.docx]

Rebutal letter.

Dear editor, dear reviewers,

Thank you for your critical reading and your supportive comments. We have addressed all the specific recommendations and revised the manuscript accordingly. Reviewers asked to show the responses of healthy donors (HD). However, although we are grateful for this comment, we did not include the data for HD in the manuscript, for a sake of clarity, as they showed similar responses to AC. We provided a figure for reviewers only showing those similar responses of AC and healthy donors.

As requested by the editorial board, we provided an author summary and a supplementary Figure 5 showing the lack of responsiveness of innate cells from frozen PBMCs. This data was stated “data not shown” in the original publication.

We hope that our significantly revised manuscript will now be suitable for publication in Plos Neglected Tropical Disease.

Sincerely,

Hélène Dutartre

**Reviewer #1:** The manuscript requires some minor editing for English usage. E.g. ‘seed’ line 36;  line 306 ‘make the bond’; line 308 ‘in this line’ etc.

Answer: Thanks, we edited the text accordingly.

Reviewer #2: (No Response)

Reviewer #3: (No Response)

**Reviewer #1:** Review of “Immunoprofiling of fresh HAM/TSP blood samples show altered innate cell responsiveness”. This manuscript evaluated innate immune responses in fresh blood samples from HAM/TSP patients, asymptomatic carriers of HTLV-1 and healthy controls. The authors stimulated whole blood with a TLR7/8 agonist and evaluated the production of IFN-a, IFN-g, MIP1a, TNF-a and IL-12 by dendritic cells (DCs), monocytes and natural killer (NK) cells by intracellular cytokine staining coupled with detailed immunophenotyping

The authors observed that the frequency of DCs, monocyte and NK cell subsets was broadly similar in all study participants. Intermediate monocytes and cDC2 were present at higher frequencies in HAM/TSP patients, and CD56highCD16- NK cells were present at lower frequencies in HAM/TSP patients. There were also several notable differences in cytokine production between ACs and HAM/TSP patients: HAM patients had higher frequencies of IL-12+pDCs, IL-12+cMonocytes, MIp1a+IntMonocytes, IL-12+ncMonocytes and IFN-g+CD56highCD16+ NK cells than ACs, and lower frequencies of IFN-a+cDCs, TNFa+cDCs, IFN-a+cDC2s, MIP-1a+ cDC2s, MIP-1a+cMonocytes and TNFa+CD56highCD16+ NK cells.

The authors propose that increased production of cytokines in response to TLR7/8 stimulation observed in HAM/TSP patients might be linked to the neuroinflammation in HAM/TSP patients, and the reduced responsiveness of DCs and monocytes could represent incomplete repression of innate immune responses by corticosteroid treatment in HAM patients.

The paper is well written and would be of interest to those studying HTLV-1 pathogenesis, innate immune responses in retroviral infection and inflammatory disease.  HAM/TSP is an understudied consequence of HTLV-1 infection, and this study addresses the important question of whether innate immune responses are dysregulated in HAM/TSP patients. Due to the nature of the cohort available (all HAM/TSP patients studied had received corticosteroid treatment), it is impossible to determine whether the observations relate to treatment or disease status. However, the cohort of patients is described comprehensively, experimental techniques and data are clearly presented and appropriately interpreted. Furthermore, the data highlight that evaluation of fresh samples is essential in investigating innate immunopathogenesis in viral infection, and publication of this manuscript will justify future much-needed studies.

Answer: We thank the reviewer for their nice comments and support of our work.

Minor concerns

Monocyte gating data is missing from S fig 1, NK gating differs in fig 4a and S fig 1. NK cytokine gating data missing is from S fig 2.

Answer: We apologize for these missing information and misunderstanding. Monocytes gating was included in Figure 3A. We apologized, indeed the gating presented in Figure 4 for NK was not correct. We thus remove this gating from Figure 4 as it was presented in Sup Figure 1. We added cytokine gating for NK in S fig 2. The text was modified accordingly.

Do the t-SNE and boolean analyses presented in fig 2/3/4 represent data from an individual patient from each group, or were all patients included?

Answer: t-SNE and Boolean analyses were performed using data from all the samples of a clinical group. Thus the data are corresponding to the pool of these data (i.e. pool of 15 HD samples vs pool of 15 AC samples vs pool of 15 HAM/TSP samples. We specify this in the Material & Methods page 6 line 182 and page 8 line 244.

The discussion refers to predictive power for future progression, however longitudinal data was not presented. E.g. line 358/359

Answer: The reviewer is right, we couldn’t perform longitudinal analysis of these samples. We thus clarify the discussion and the sentence in line 362 is now “Our first objective was to identify potential immune biomarkers that could sign the disease before symptomatic manifestations”. Indeed, we expected to retrospectively analyzed the samples in case some of the AC evolved toward HAM/TSP several years after the sampling since, individuals of the cohort are still followed by Dr Casseb at Institute of Infectious Diseases “Emílio Ribas” (IIER). Although some showed signs of inflammation that could evoke evolution toward HAM/TSP (stated in line 431 of the manuscript) no difference in phenotype or function of innate cells were detected when compare to AC that didn’t evolve. However, we believe this study could serve as a reference and foster longitudinal sampling of AC, which is to date a very challenging task.

Similarly, the line ’Sociodemographic factors, PVL or innate cell frequencies do not predict a HAM/TSP progression’ (line 181-182) should be modified to reflect the results presented.

Answer: We modified the heading “ Sociodemographic factors, PVL or innate cell frequencies are similar in AC and HAM/TSP. » in page 6 line 188 to avoid misunderstanding based on longitudinal analysis that was not perform in this study.

**Reviewer #2:** Rocamonde, et al. investigated cytokine responses of dendritic cells and monocytes upon stimulation with TLR7/8 stimulation in patients with HAM/TSP and compared them to those of ACs.

Despite the inflammatory disease manifestation, the frequency of IFNa and MIP1a-producing DCs in HAM/TSP patients was lower than ACs. In contrast, IL-12 and MIP1a responses in the monocytes were higher in HAM/TSP patients. This study demonstrated the different inflammatory responses upon TLR7/8 stimulation between HAM/TSP patients and ACs. However, since all the HAM/TSP patients evaluated were under treatment, it is difficult to judge whether the differences demonstrated could be a consequence of corticosteroid treatment or representing disease-specific response. In addition, the cytokine responses against TLR7/8 stimulation in seronegative controls were not shown. Although this study includes potentially important information, several points should be improved.

Answer: We thank the reviewer for their critical reading and suggestions to improve our finding.

Major points
1.	The authors speculated that the reduction of innate immune response in DCs could be a consequence of corticosteroid treatment. Indeed, corticosteroid is known to suppress inflammatory cytokines. However, in this study, they showed discrepancies between IFNa/TNFa and IL-12/IFNg responses and also between the responses of DCs and monocytes. It may be difficult to examine the responses in HAM/TSP without treatment, but the authors should at least assess the cytokine responses to TLR7/8 in DCs and monocytes from seronegative individuals in the presence or absence of corticosteroids in vitro.

Answer: We acknowledge the suggestion of the reviewer. We did address the response of innate cells form seronegative healthy donors upon stimulation with TLR7/8. The results were similar to that of AC, shown here for the reviewer only. Because we were focused on the difference in innate responsiveness induced by the clinical manifestation and because the presence of the virus with disease didn’t show any difference (compare HD and AC), we choose to not show results from the healthy donors in the manuscript. We agree that comparison of innate cell responsiveness in presence or absence of corticosteroids would be very helpful to strengthen our finding. However, this experiment would be more accurate if performed using samples from AC, in which the virus is present, using different concentration of corticosteroids and kinetics of treatment. For practical reasons, and because it is very difficult now to travel to Brazil, to sample AC and to perform the experiment on site, we are sorry not to be able to provide this kind of control.

2.	The finding of enhanced IL-12 response in HAM/TSP patients is interesting and potentially important. However, it is curious that the authors described this inflammatory cytokine as an anti-inflammatory cytokine (line #302-303) but did not examine a representative anti-inflammatory cytokine IL-10. Is there any data on IL-10 response?

Answer: This is an interesting point. We do not have data on IL-10 unfortunately. Sorry for the mis writing of IL-12 as anti-inflammatory. We corrected the text accordingly page 10 line 311.

3.	Correlation between IPEC score and the frequency of DCs positive for IFNa, TNFa or IL-12 was shown in Suppl. Fig. 3, although it was not statistically significant. As the authors clearly showed enhanced MIP1a and IL-12 responses in HAM/TSP monocytes, correlation between the monocyte responses and IPEC score should be shown also.

Answer: We acknowledge that correlations are not significant. We hypothesized that it may be relative to the sample size. We are planning additional studies in the future to address this question using larger number of patients, especially those at early phase of disease, as shown previous by our group (Haziot et al. 2018). We didn’t observe any correlation between IPEC and IL-12 or MIP1A monocytes responses as presented below for reviewer only. We modified the text accordingly page 13 line 403.

**R^2^ = 0.004**

*p-value = 0.82*

**R^2^ = 0.001**

*p-value = 0.89*

**R^2^ = 0.005**

*p-value = 0.79*

Minor points
1.	Suppl. Table 1. Does ‘Treatment’ mean corticosteroid treatment in all the patients shown? It should be clearly described about the use of corticosteroid in the column of ‘Treatment.’

Answer: Our study was performed at least 15 days before or after pulse therapy with IV methylprednisolone. We included this information in the text page 3 line 100 in the Material & Method section. We don’t have more specific information about the treatment for each patient.

2.	Suppl. Table 1. What is the purpose to show ‘breastfeeding’ without information of HTLV-1 infection in mothers and ‘familiar situation’ with marital status? These may be confusing.

Answer: Thank you for the comment. We remove this information.

**Reviewer #3:** Rocamonde et al. analyzed innate cells including dendritic cells, monocytes, and NK cells from HAM/TSP patients using fresh blood samples and compared them with those from HTLV-1 carriers. They found some dysregulation of these immune cells from HAM/TSP patients, high frequencies of MIP-1a producing monocytes, IL-12 producing pDC, and IFN-g producing NK cells. The study comprehensively profiled the innate cells in HAM/TSP, however, several issues need to be addressed before publication.

Answer: We thank the reviewer for their critical reading and suggestion to improve our finding.

Major points;

1.	In line 203-205, this sentence is an interpretation of the data and should be moved to the Discussion.

Answer: Thanks, we move the comments in discussion as recommended.

The authors found no differences in the PVL between HAM/TSP patients and HTLV-1 carriers, which is controversial to the previous reports from many research groups. Please discuss this discrepancy in the Discussion. Is there a possibility that the treatment affected the PVL or motor disability?

Answer: We agree with the reviewer that our results are not in concordance with reports from other groups. We although observed an increased tendency of the mean of PVL in HAM/TSP that did not reach significance, probably because of high dispersion of PVL from AC. To our knowledge, literature report no information regarding the effect of HAM/TSP treatment on PVL. Because we didn’t perform longitudinal analysis we cannot answer whether the corticoid treatment affect PVL or motor disability. Furthermore, our point was to stress that the absolute PVL is not per se an indicator of disease or disease evolution because we observed in our cohort diagnosed HAM/TSP patients with low PVL and diagnosed HAM/TSP patients with PVL similar to that of AC. We added this in the discussion page 12 lines 392-394.

2.	In line 259, they suspected that the low responsiveness in cytokine is due to the corticosteroid treatment. If so, this study may not reflect the responses of innate immune cells in the natural course of the disease. I strongly recommend that they collect blood samples from untreated HAM/TSP patients and analyze them, especially if they are looking for a predictive immune marker for progress towards HAM/TSP from carrier state.

Answer: This is a very good point that we would like to perform in the future. However this is a very challenging task as it is very difficult to collect samples from HAM/TSP patients before their treatment (the protocol need to be implemented in the clinic) and furthermore to have sufficient fresh blood samples to analyze innate cell responsiveness that is lost after freezing.

3.	In Figures 2 to 4, they detected cytokine-producing cells by flow cytometry and compared the frequency in each cell population. However, the amount of the produced cytokines from each cell is not clear to be the same, I recommend measuring the amount of cytokines in the culture supernatant by ELISA or use the mean fluorescence intensity reflecting the cytokine amount of the cells.

Answer: We didn’t perform ELISA on supernatant because we wanted to have data at the single cell level. We provided the results using MFI as supplementary Figure 4A and B. It strengthened the decrease in TNFα production by cDC2 and pDC and IFNα in pDC. Thus, both cell frequencies and amount of cytokine produced are decreased in HAM/TSP patients after stimulation. We modify the manuscript accordingly in page 8 lines 254-258 and page 10 line 311.

The authors found that IL-12-producing DC and monocytes were high frequency in HAM/TSP patients. It is known that IL-12 stimulates NK cells. Do the increased IL-12 affect the NK cell function and modify the clinical status of HAM/TSP patients?

Answer: We agree with the reviewer and searched for correlation between the amount of IL-12 and NK function. The result are presented in Figure 4F for IL12 in ncMono and pDC and IFNγ in NK CD16^-^ although we observed a tendency, the correlation didn’t reach statistical significance. We showed correlations with IPEC Score in S Fig. 3

Minor points;
1.	In line 121, 10 to the 6th power may be 10 to the 5th power.

Answer: Thanks for the critical reading. We corrected accordingly.

2.	In supporting figure 1 and line 155, the CD56highCD16- cell population contains CD56dimCD16- cell population, but in Figure 4A, the cell population did not. Please correct the supporting figure 1.

Answer: We apologize for these missing information and misunderstanding. Monocytes gating was included in Figure 3A. We apologized, indeed the gating presented in Figure 4 for NK was not correct. We thus remove this gating from Figure 4 as it was presented in Sup Figure 1. We added cytokine gating for NK in S fig 2.

3.	In line 202, “independently of their clinical status and sex” is inappropriate because the data included all samples but is not divided by these attributes and compared among subgroups.

Answer: The reviewer is right, the correlation included all samples. However, we indicated in figure 1C the status of each samples by a color code and a symbol code. It shows that female (coded in red) are not clustered from male (coded in blue) neither are AC (coded with a circle) from HAM/TSP (coded with a triangle).

4.	In line 212, did they stimulate only the blood from HTLV-1 carriers?

Answer: We included healthy donor in the sampling and analysis. However, we didn’t show the results for a sake of clarification. We observed that HD behave similarly to AC and choose not to present their data to focus on the differences innate cell responsiveness of HTLV-1 infected individuals and patients to identify potential drivers of clinical symptoms.

5.	In line 225, MIP-1 alpha is a more general expression than Mip-1 alpha.

Answer: Thanks. Corrected accordingly.

6.	In Figure 3A, they defined the classical monocytes as CD16 negative but positive for CD3/14/15/19 cocktail. I think monocytes do not express either CD3 or CD19. Monocytes were firstly gated from HLA-DR+CD11c+, suggesting that this classical monocyte population did not contain T cells and B cells. Why did not the authors use a CD14 antibody instead of the antibody cocktail?

Answer: The antibody panel was designed and optimized to analyze both innate and adaptive immune responses, although we presented here only results from innate responses. For a sake of constraint expenses, blood samples were stained with the “lin” panel to identify or exclude lineage cells as B; T; granulocytes or monocytes and then samples were divided in two and stained either for innate cells (CD56; CD11c; CD16; BDCA1; BDCA2; BDCA3; HLADR) or for adaptive cells ( CD45RA, CD4, CD8, TCRg9) before specific intracellular staining (IFNα, IL12, TNFα IFNγ for innate cells and IFNγ, IL17a, IL2, IL21 and TNFα for adaptive cells)

7.	Please explain why the authors divided the NK cells into two subsets, CD56dim and CD56high.

Answer: NK cells are recognized as a heterogenous population based on the expression of different receptors. Classically NK cells are divided in CD56^dim/low^ or CD56^high^ based on their functions: CD56^low^CD16^high^ NK cell subset expresses high levels of perforin and mediates natural and antibody-dependent cellular cytotoxicity, and enhanced killing while CD56^high^CD16^±^ NK cells are characterized by low levels of perforin, and are primarily specialized for cytokine production. We anticipated to observed higher expression of IFNγ by CD56^high^CD16^±^ NK cells from HAM/TSP patients because of the inflammatory status of the disease and we were expecting that corticoid treatment was expected to control. We specify the reasoning in the text page 10 line 320-326.

8.	In line 394, the increased IFN-g production of NK cells was induced by R848 stimulation in vivo. Why can the authors argue that corticoid treatment failed to control the production of inflammatory cytokines?

Answer: The stimulation were performed *in vitro* on freshly collected samples to address innate cell responsiveness in infected HAM/TSP patients under treatment to that of infected individuals with no clinical symptoms. Corticoids are thought to block the production of inflammatory cytokines, including TNF-α, IFN-γ IFN-α. Because NK response were similar in HAM/TSP patients under treatment and in infected individuals and because we observed higher frequencies of dendritic cells and monocytes producing TNF-α, or IL-12 in samples from HAM/TSP patients after stimulation, we conclude that corticoids failed to control the production of inflammatory cytokines by several innate cells.

--------------------
