# Supplementary material for: Immunoprofiling of fresh HAM/TSP blood samples shows altered innate cell responsiveness
Source: PLoS Negl Trop Dis. 2021 Nov 12;15(11):e0009940. doi: 10.1371/journal.pntd.0009940 (PMC8631667; doi:10.1371/journal.pntd.0009940)
Supplement: Attachment — Submitted filename: Rebutal october.docx [file pntd.0009940.s010.docx]

Dear editor, dear reviewers,

Thank you for your new deep critical reading. We have addressed the new specific recommendations from reviewer #3 and revised the manuscript accordingly. Specifically, we removed the search for predictive marker as the purpose of this study, and dampened our point that corticoid treatment failed to control inflammation. We would like to thank Reviewers #1 and #2 who acknowledge our responses and recommended publication. We agree that the overall clinical significance remain obscure but we believe that our observations would encourage new study using larger cohorts to decipher the role of innate cell function in HAM/TSP and the efficiency of corticoid treatment at reducing inflammation supported by innate cells.

We hope that our significantly revised manuscript will now be suitable for publication in Plos Neglected Tropical Disease.

Sincerely,

Hélène Dutartre

Reviewer #1: All of my concerns have been addressed and I recommend publication of the manuscript.

Reviewer #2: In the revised manuscript, the authors made several modifications to improve the clarity. However, they could not add any data about the effect of corticosteroids. It is unfortunate that the clinical significance of the observations remains obscure. Nevertheless, this study contains valuable data of fine immunological analysis using rare clinic samples.

Reviewer #3: Rocamonde et al. responded well to my comments. However, there are still some points to be improved. Followings are new comments corresponding to the previous comment numbers.  

Major points;

1. The authors found no differences in the PVL between HAM/TSP patients and HTLV-1 carriers, which is controversial to the previous reports from many research groups. This point is important. In the point-to-point reply, the authors try to explain why the discrepancy occurs. I recommend writing like this in the Discussion section.

Answer: We modified the discussion as suggested to precise why we found no differences in the PVL between HAM/TSP and HTLV-1 carriers. Please see lines 406-414.

2. I understand how difficult to collect fresh blood samples from pretreatment patients. Lines 24 and 42 imply that the purpose of this study is to find predictive immune markers for HAM/TSP. However, the study using samples from patients with corticosteroid treatment can find the differences in innate immune cells between HAM/TSP and ACs but would not find prediction markers from ACs to HAM/TSP.  Please modify lines 24 and 42.

Answer: We modified lines 24 and 42 and remove suggestions that our study would find predictive immune markers. Line 24 should now be read as “Immune cell from the adaptive compartment are involved in disease manifestation but whether innate cell functions participate in disease occurrence has not been evaluated.” And line 42 as “inflammation has been linked to the deregulated activation of T-cells, but the role of innate cells has not been investigated yet.” Please see lines 24-26 and 47-48.

Minor points;

3．I disagree with the author's claim. They included all samples to investigate whether PVL correlates with age. However, for example, female HAM/TSP (red triangles) do not seem to correlate.

Answer: To our knowledge there is not a justified criterion to exclude this group of patients from the analysis. Since in our cohort PVL is not statistically significant in HAM/TSP and there is no possibility to predict HAM/TSP based on PVL, we wondered whether it would exist a correlation between PVL and age. The correlation of PVL and age is not completely meaningless biologically, since factors such as exposition to repetitive viral infection with time or defective/diminished immune system in elder people could be a factor influencing the onset of the HAM/TSP. Our aim was to make this data/type of analysis available for consideration in future studies.

8. To know whether corticosteroids failed to control the production of inflammatory cytokines, the data that compare the production in HAM/TSP with corticoids or without those are needed.

Answer: We agree with this comment and it would be of interest. However, this would require a complex longitudinal study. This is a very challenging task due to the low number of HAM/TSP patients diagnoses and the logistical difficulty of collecting fresh blood samples from HAM/TSP patients before their treatment (the protocol would then need to be implemented in the clinic), and perform the experiments right away since innate cell responsiveness is lost after freezing. We are sorry not to be able to provide this kind of control and we removed the suggestion that corticoid treatment failed to control inflammation in the revised manuscript (line 101 in the introduction; line 457 in the discussion).
